# Effective inactivation of *Saccharomyces cerevisiae* in minimally processed *Makgeolli* using low-pressure homogenization-based pasteurization

**DOI:** 10.1186/s40064-015-0936-4

**Published:** 2015-04-02

**Authors:** Jin Seop Bak

**Affiliations:** Department of Chemical and Biomolecular Engineering, KAIST, 291 Daehak-ro, Yuseong-gu, Daejeon 305-701 Republic of Korea; Institute of Advanced Machinery and Design, Department of Mechanical and Aerospace Engineering, Seoul National University, 1 Gwanak-ro, Gwanak-gu, Seoul 151-744 Republic of Korea

**Keywords:** Low-pressure homogenization-based pasteurization, *Makgeolli*, Minimal processing-preservation, *Saccharomyces cerevisiae*, Suspension stability

## Abstract

In order to address the limitations associated with the inefficient pasteurization platform used to make *Makgeolli,* such as the presence of turbid colloidal dispersions in suspension, commercially available *Makgeolli* was minimally processed using a low-pressure homogenization-based pasteurization (LHBP) process. This continuous process demonstrates that promptly reducing the exposure time to excessive heat using either large molecules or insoluble particles can dramatically improve internal quality and decrease irreversible damage. Specifically, optimal homogenization increased concomitantly with physical parameters such as colloidal stability (65.0% of maximum and below 25-μm particles) following two repetitions at 25.0 MPa. However, biochemical parameters such as microbial population, acidity, and the presence of fermentable sugars rarely affected *Makgeolli* quality. Remarkably, there was a 4.5-log reduction in the number of *Saccharomyces cerevisiae* target cells at 53.5°C for 70 sec in optimally homogenized *Makgeolli*. This value was higher than the 37.7% measured from traditionally pasteurized *Makgeolli*. In contrast to the analytical similarity among homogenized *Makgeollis*, our objective quality evaluation demonstrated significant differences between pasteurized (or unpasteurized) *Makgeolli* and LHBP-treated *Makgeolli.*

Low-pressure homogenization-based pasteurization, *Makgeolli*, minimal processing-preservation, *Saccharomyces cerevisiae*, suspension stability.

**Electronic supplementary material:**

The online version of this article (doi:10.1186/s40064-015-0936-4) contains supplementary material, which is available to authorized users.

## Introduction

*Makgeolli*, also known as Takju, is a traditional rice-based unfiltered wine in Korea. As a result of the open program associated with the *Makgeolli* brewing process, various microorganisms such as yeasts (*Candida, Hansenula, Pichia, Saccharomyces,* and *Torulopsis* spp.), fungi (*Aspergillus*, *Mucor*, and *Rhizopus* spp.), and bacteria (*Aerobacter, Bacillus, Lactobacillus, Leuconostoc, Micrococcus,* and *Pseudomonas* spp.) organically coexist in unpasteurized *Makgeolli* (Kim et al. 2010; Lee and Rhee 1970). During distribution and ageing, spoilage and biodegradation of *Makgeolli* quality is frequently induced by these microorganisms, resulting in the generation of off- flavors (especially due to formation of diacetyl compounds) and an acidic content (Park et al. 2013). Therefore, systematic control of the microbial population throughout the manufacturing process is crucial for improving the *Makgeolli* commercial shelf life.

Thermal treatment is one of the most broadly used preservation processes for food, and an economical and efficient strategy for inactivation of major microorganisms often found in commercial *Makgeolli*. Due to thermal resistance of target organisms such as fermentable yeasts and lactic acid bacteria, effective treatment conditions such as temperature and time have been extensively studied in order to support practically optimized pasteurization. In an applied platform, unpasteurized *Makgeolli* is generally subjected to two systems: a long program (mainly 10–15 min at 55–65°C) and a short program (typically 23 sec at 80°C) to generate a commercially stable shelf life of 14–50 days (Park et al. 2013; Lee et al. 1991; Bae et al. 1990). Recently, in order to prevent critical physicochemical problems such as discoloration, sedimentation, and off-flavors, an end user definitely has a preference for the long program. However, there are still far too many limitations such as incomplete sterilization, which affect commercial value.

This study therefore aims to find alternatives to the high temperature preservation system by investigating a low temperature platform in a short program. Interestingly, because the turbid suspended colloids in *Makgeolli,* observed when the filtration process is not performed, are based on random dispersion patterns (Haard et al. 1999), excess heat for adequate sterilization may be inevitably required for the *Makgeolli* thermal process. Application of the homogenization process may provide a reasonable alternative to the thermal process. The homogenization action in colloidal systems involves the uniformity (or reduction) of particle size, which is obtained by the action of shearing forces (Kiełczewska et al. 2003). No previous studies have applied thermal treatment to homogenization, especially at low pressure without any side effects, in order to verify the major factors in microbial inactivation of fermentation-based colloidal systems such as *Makgeolli*. Furthermore, the use of unsystematic low-pressure homogenization-based pasteurization (LHBP), based on both size optimization (especially by no filtration alcoholic beverages) and target pasteurization (by selection of target microorganisms), to enhance the conserved stability of biochemical sensory properties; has not been sufficiently examined for any commercial program to date.

This study was conducted to verify the efficiency and feasibility of the LHBP program. Its impact was evaluated based on various biochemical properties including microbial inactivation and sensorial quality of LHBP-treated *Makgeolli*. An optimized LHBP-based methodology under mild conditions could lead the way for bioactive preservation and improved *Makgeolli* shelf life, while also meeting the demands of the consumers for fresh quality wine and this therefore further raise our competitiveness in the world market.

## Materials and methods

### Sample preparation

Commercial *Makgeolli* products made of rice, with an ethanol concentration of approximately 7%, were purchased from local markets, and then stored at 4°C for half a day until use. Prior to the experiment, the initial acidity of the products was routinely determined in order to continuously maintain the same condition. Prior to pasteurization, *Makgeolli* samples were homogenized using the 1500KL24 5RA system (Schier Company Inc., Kiefer, OK) at 20°C in order to maintain a uniformed state for colloidal stability and even distribution of particle size. All samples were divided into six parts and homogenized once or twice at 15.0, 25.0, or 35.0 MPa. The homogenized samples were stored at 4°C for 1–2 h until use. No homogenization was applied to the control samples.

### Physicochemical analysis of colloidal stability

According to a previously reported method with minor modifications (Priepke et al. 1980), suspension stability was confirmed by monitoring the top to bottom ratios of total insoluble-solids in *Makgeolli* samples stored undisturbed in a 20 ml bottle for 3 days at target temperatures of 4, 25, and 37°C. After gravity sedimentation at steady state, the supernatant portion from all samples was analyzed for dissolved total solids using a drying method at 105°C. In addition, the apparent viscosity of the samples was determined at target temperatures with an LVDV-I+ rotational viscometer (Brookfield Engineering Laboratories Inc., MA) using an LV-1 cylindrical spindle. Data was collected based on 2 min at 60 rpm after the spindle was immersed, to consider the effect of time dependency and thermal balance. Furthermore, after homogenization pretreatment, the relative diameter of insoluble particles in *Makgeolli* samples was measured using a SALD-7100 particle analyzer (Shimadzu Co., Ltd., Kyoto, Japan). Lastly, in order to visualize an entire dispersion pattern, a microscopic image of target samples was examined by the Image Pro-Plus system (Media Cybernetics, Bethesda, MD). All physicochemical analyses were systematically conducted in triplicates.

### Pasteurization process and data analysis

Following homogenization treatment, the pasteurization of processed *Makgeolli* samples was conducted using the tubular-type heat exchanger (THE) system with minor modifications based on the custom fitting service (Daihan Scientific Co., Ltd., Seoul, Korea). The details of the physical properties regarding the THE system for low-pressure homogenization-based pasteurization (LHBP) are shown in Additional file 1 (especially Figure S1 and Table S1). Regardless of the homogenization condition (whether performed once or twice), target sample was systematically subjected to the THE system and maintained at 50.5, 53.5, and 57.5°C, respectively. The samples were fully circulated under hot water, with the heating time ranging from 5 to 70 sec (except for holding and cooling times), depending on target heating temperatures. All pasteurization treatments were terminated by immediately placing the LHBP-treated samples into an iced water bath. Data collection from the LHBP program was simultaneously recorded using the 2625A Data Logger (John Fluke Mfg. Co. Inc., Everett, WA).

Microbial thermal inactivation studies were repeated at least 6 times for each time/temperature combination and all of the plate counts of targets (yeasts and bacteria) were conducted in triplicates. The *D*-values (thermal death point or index as decimal reduction time) of yeasts in LHBP-processed *Makgeolli* were predicted from survival curves (based on linear regression), which were calculated by plotting the log microbial counts (i.e., log CFU/g) against heating time for each temperature (i.e., 50.5, 53.5, and, 57.5°C). The statistical significance of the thermal death index was determined based on a *t*-test and ANOVA analysis using SAS ver. 9.2 (SAS Institute, Cary, NC) and SigmaStat 3.5 (Systat Software, San Jose, CA).

### Identification of target microorganisms

The DNA fingerprinting of yeast strains in *Makgeolli* was identified using simplified randomly amplified polymorphic DNA (RAPD) analysis, which is based on direct PCR methods with minor modifications (Bautista-Muñoz et al. 2003; Lockhart et al. 1997). Yeast cells (as fermenting/aging index) from *Makgeolli* samples were harvested by centrifugation at 12,000 rpm for 10 min. After discarding the supernatants, the fractions were suspended in TE buffer containing 40 mM EDTA and 100 mM Tris–HCl at 25°C and stored at −80°C until use. In order to obtain genomic DNA, target yeasts were grown on malt extract agar (Difco, Detroit, MI) at 25°C for approximately 48 h (or below 60 h). Considering the peculiar property of yeasts to initially abundantly proliferate, (i.e., within approximately 3 days under the commercially open platform), after immediately performing minimally processing treatment (either LHBP or only homogenization), the yeasts were counted using a generally confirmed standard plating method. For reference, after the stepwise dilution, all identification experiments were conducted based on twenty biological replicates. A single colony from the plate was subcultured at 25°C for 48 h on yeast extract-peptone-dextrose broth (Difco), and then target DNA was extracted by a previously confirmed method (Lehmann et al. 1992). Regarding the DNA quality, A_260_/A_280_ ratios of 1.8 to 2.1 were determined using a spectrophotometer (UVmini-1240, Shimadzu Corp., Kyoto, Japan). To determine genetic variation, briefly, a RAPD system was performed where a mixture of genomic DNA [20 ng], the appropriate primer [0.4 μM], dNTP [200 μM), and Taq DNA polymerase [1.2 U] were mixed with a PCR buffer (10×) provided by the manufacturer (Takara Bio Inc., Otsu, Japan). These patterns were obtained using the primer (10-mers) OPA-18 (5′-AGCTGACCGT-3′) (Bioneer Corp., Daejeon, Korea). The PCR conditions were: 38 cycles i.e., 1 min at 94°C, 1 min at 36°C, and 2 min at 72°C. In order to ensure accurate identification, the metabolic capabilities of various carbon sources were examined by the API 20C AUX index (bioMérieux SA, Marcy-l’Etoile, France). Further details are provided in Additional file 1.

### Quality evaluation based on key biochemical characteristics

The changes in intramolecular parameters, such as alcohol, acidity (as determined by alkalimetric titration), bacterial cells, the presence of fermentable (reducing) sugars, and amyloglucosidase, in minimally processed *Makgeolli* were analyzed according to the standard analytical methods of the National Renewable Energy Laboratory (http://www.nrel.gov/biomass/capabilities.html). In extracellular amyloglucosidase, a mixture containing both 1.0 mL of diluted broth supernatant and 1.0 mL of 1–2% starch solution in 0.05 M sodium citrate buffer (pH 4.8) was reacted at 30°C for 30 min, and then reducing sugars were checked by the 3,5-dinitrosalicylic acid method at 540 nm. For reference, the international unit (IU) of an enzyme was defined as the amount of enzyme that releases 1 μmol of reducing monomer per minute. Regarding the activation of acidity after the LHBP program, the bacterial cells in processed *Makgeolli* were serially diluted in Luria Bertani (LB) medium (Difco), and then plated on LB agar plates containing 50 μg/ml nystatin (Sigma-Aldrich, St. Louis, MO) which were incubated for 24 h at 37°C.

Based on a previously reported protocol (i.e., extracellular metabolic profiling; Bak 2014), downstream odor-active chemicals, as classified by the National Institute of Standards and Technology database (http://webbook.nist.gov/chemistry/), in *Makgeolli* suspensions were analyzed using a quadrupole 5975/7890 mass analyzer (Agilent Technologies, Waldbronn, Germany) with DB-5MS column (J&W Scientific, Folsom, CA). After preprocessing for mass spectrometry (Additional file 1), based on a previously confirmed procedure with minor modifications (Bak 2014; Park et al. 2013), the GC-MS profiles were as follows: 40°C for 5 min, 5°C/min to 130°C, 15°C/min to 220°C with holding for 1 min, 2.5°C/min to 265°C with holding for 1 min, 10°C/min to 280°C with holding for 1 min, 1°C/min to 290°C, and 10°C/min to 300°C. Further details are provided in Additional file 1. After eight experiments, the significance of inducible differences for the chemicals in each sample was analyzed using the paired *t*-test. The changes between samples were checked using the unpaired *t*-test and ANOVA analysis. The statistical bias for metabolome data was confirmed using SAS ver. 9.2 and SigmaStat 3.5. Lastly, the color change in LHBP-processed *Makgeolli* was measured by a JP7200F color difference meter (Juki Corp., Tokyo, Japan) at 10°C based on a previously reported methodology (Hinds et al. 1997). The ANOVA was checked, and inducible gap among groups was confirmed by Duncan’s multiple-range test using SAS.

## Results and discussion

### Colloidal stability in homogenized *Makgeolli*

**Figure 1:**
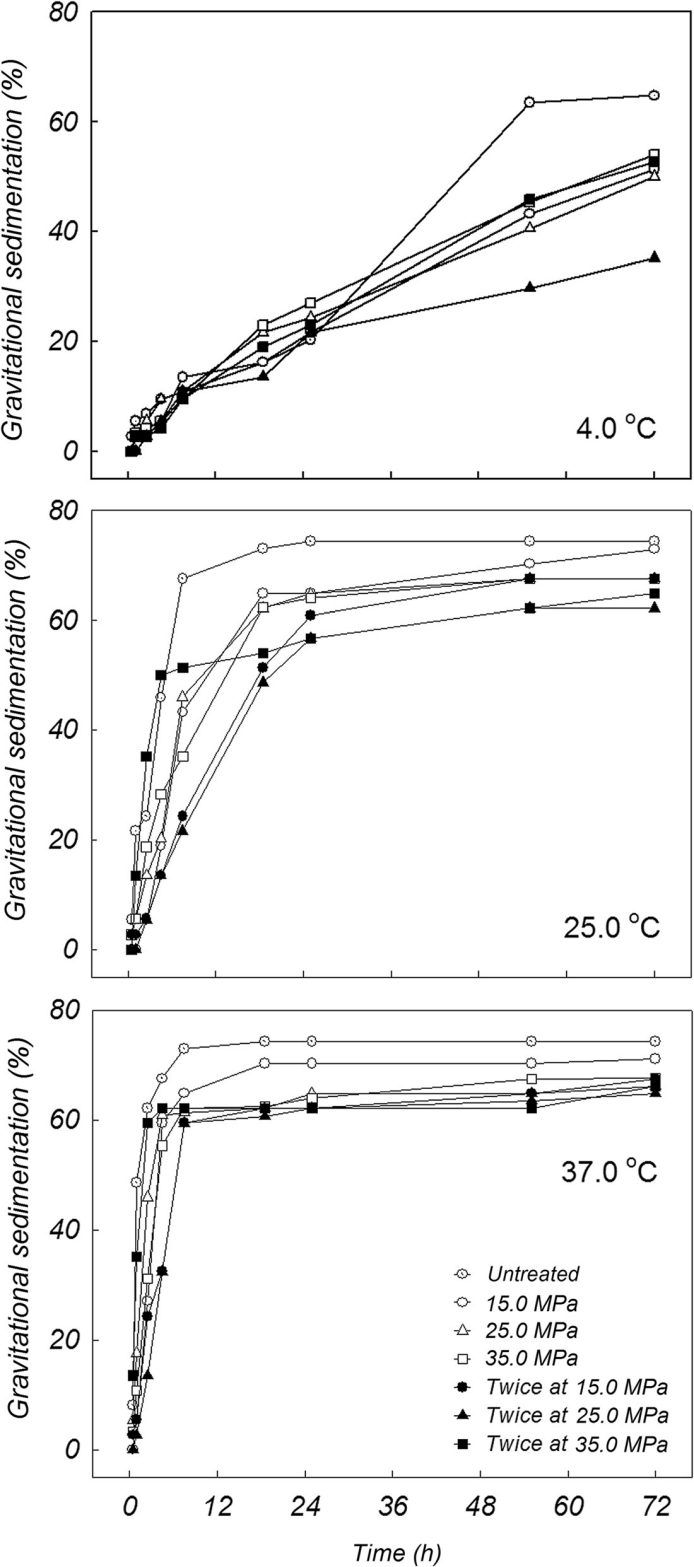
**Real-time changes of suspension stability in minimally processed** ***Makgeolli*** **samples under various homogenization conditions during the storage period.** All points shown are the mean values of triplicate experiments.

**Figure 2:**
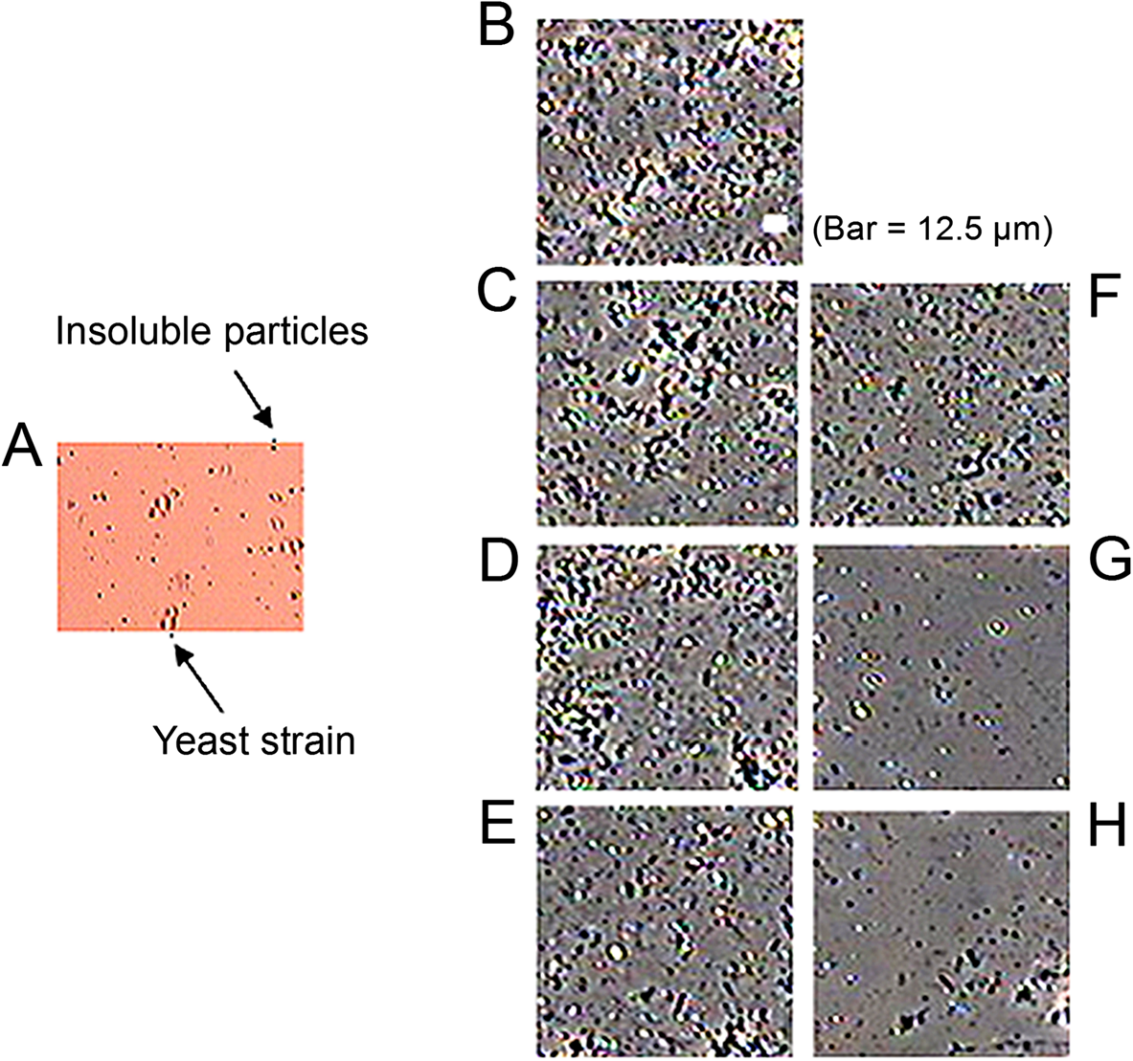
**Conserved photomicrographs of** ***Makgeolli*** **suspension pretreated by homogenization. (A)** Supernatant liquid after sedimentation (100× magnified). **(B)** Untreated (100× magnified). **(C)** Homogenized at 15.0 MPa (100× magnified). **(D)** Homogenized at 25.0 MPa (100× magnified). **(E)** Homogenized at 35.0 MPa (100× magnified). **(F)** Homogenized twice at 15.0 MPa (100× magnified). **(G)** Homogenized twice at 25.0 MPa (100× magnified). **(H)** Homogenized twice at 35.0 MPa (100× magnified).

**Figure 3:**
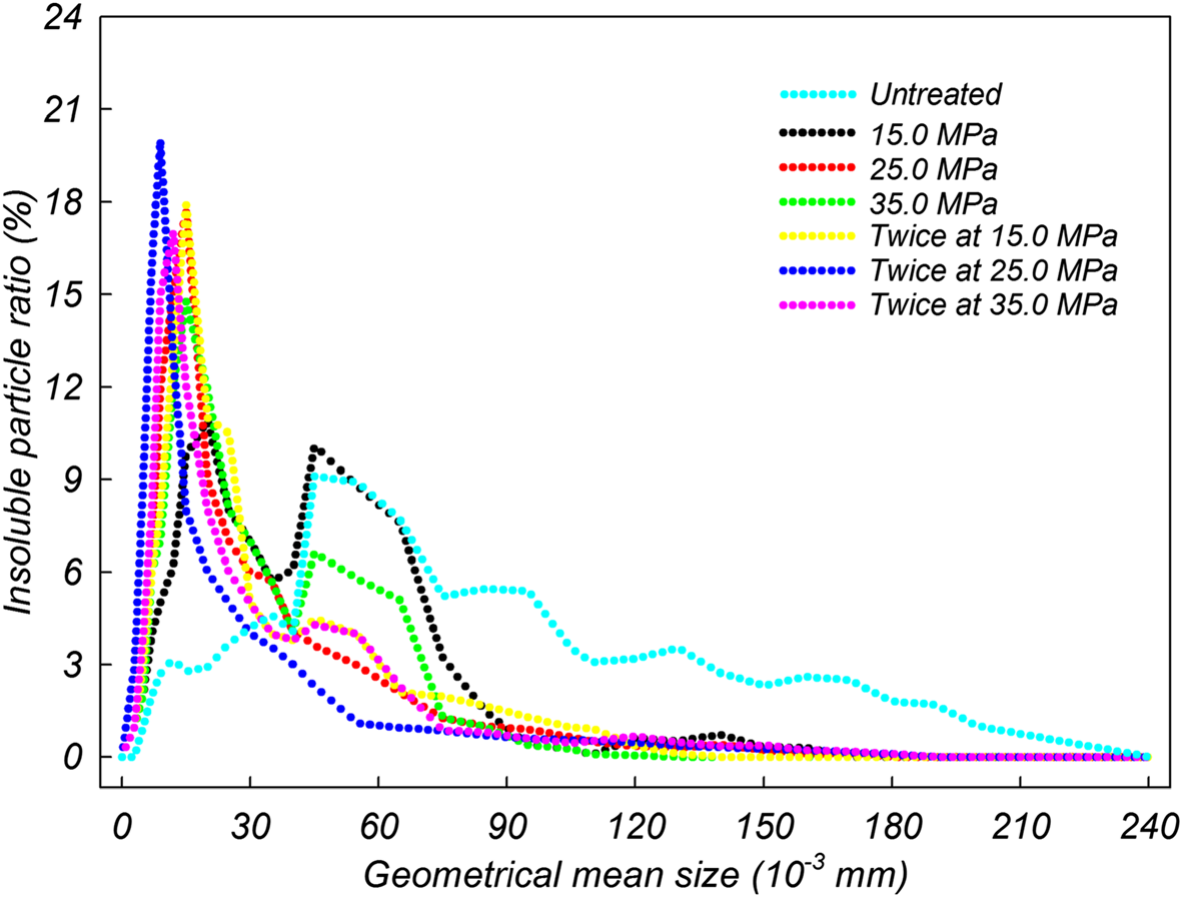
**Size distribution patterns of insoluble microparticles changed in homogenization-treated** ***Makgeolli*** **suspensions containing 7% ethanol.**

Following *Makgeolli* pretreatment during the homogenization process using mild pressure conditions, rheological stability in colloidal suspension was characterized by top to bottom ratios of total insoluble particles for 3 days (Figure 1). The homogenization level results demonstrate that the extent of increased physicochemical reaction was correlatively regulated in one direction. As storage time progressed, gravity-induced sedimentation (% theoretical maximum), which indicates the suspension stability of insoluble molecules, gradually increased regardless of storage temperature. Especially, after 72 h of sedimentation, colloidal stability from the pretreated *Makgeolli* were 65.0% and 47.3%, with commercial conditions performed twice at 25.0 MPa and at 35.0 MPa, respectively. Interestingly, pretreatment with a relatively severe dose (twice at 35.0 MPa) resulted in decreased stability, which is most likely due to substrate decomposition (or aggregation) at higher doses (Figure 2). For the colloidal suspension patterns, it was suggested that the saturating concentration, based on solubility and dispersion of large insoluble particles, of small molecules generally increases with increased homogenization pressure, except when performed twice at 35.0 MPa. Interestingly, the powerful saturating circumstances and stability may require conducting homogenizing preprocesses multiple times (e.g., twice at 25.0 MPa) rather than only in one step (e.g., once at 25.0 MPa). Furthermore, in case of a relationship between pressure parameters and multiple performance times, the difference is likely to depend on repetition level (e.g., twice at 25.0 MPa) rather than pressure strength (e.g., once at 35.0 MPa). Based on the optimal depolymerization condition (i.e., twice at 25.0 MPa), the passive convergence of molecule sizes (about < 25 μm; Figure 3) greatly affected the physical stability (especially based on layer separation) of the *Makgeolli* suspension. Therefore, the optimal dose for an effective dispersion of *Makgeolli* particles is a fixed condition of twice at 25.0 MPa.

**Figure 4:**
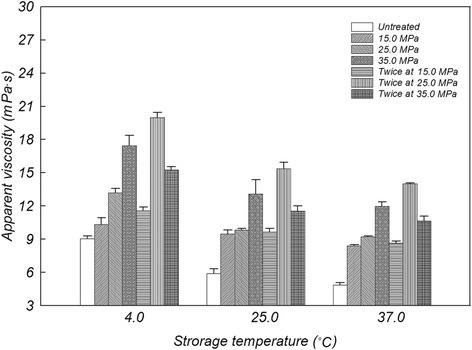
**Fluidic changes of apparent viscosity in** ***Makgeolli*** **suspension with insoluble particles under the various homogenization conditions.** All points shown are the mean ± standard deviation of observations that were analyzed in triplicate.

The samples prepared at optimal pretreatment conditions were more viscous than other groups (Figure 4), which was similar to the colloidal sedimentation patterns and independent of storage temperatures. In contrast to a previously well-known concept (Andrews et al. 1977), the current study demonstrates apparent viscosities of colloidal suspensions that were significantly affected by either mild treatment pressure or storage temperature. In the case of the homogenized cereal beverage, limited patterns of viscosity were also observed and correlated with enhanced levels of homogenization pressure (Rubico et al. 1987). This phenomenon may be closely influenced by intramolecular interaction (or solubility) among components, such as starch granules and amino acids, based on size minimization of insoluble particles due to the homogenization process. Furthermore, many peptide residues may be utilized as intracellular bridges in colloidal suspensions since hydrophilic (or hydrophobic) chains can be attributed to the formation of starch (or lipid)-protein complexes during homogenization treatment (Weegels et al. 1994).

### Microbial inactivation in minimally processed *Makgeolli* using the LHBP program

**Figure 5:**
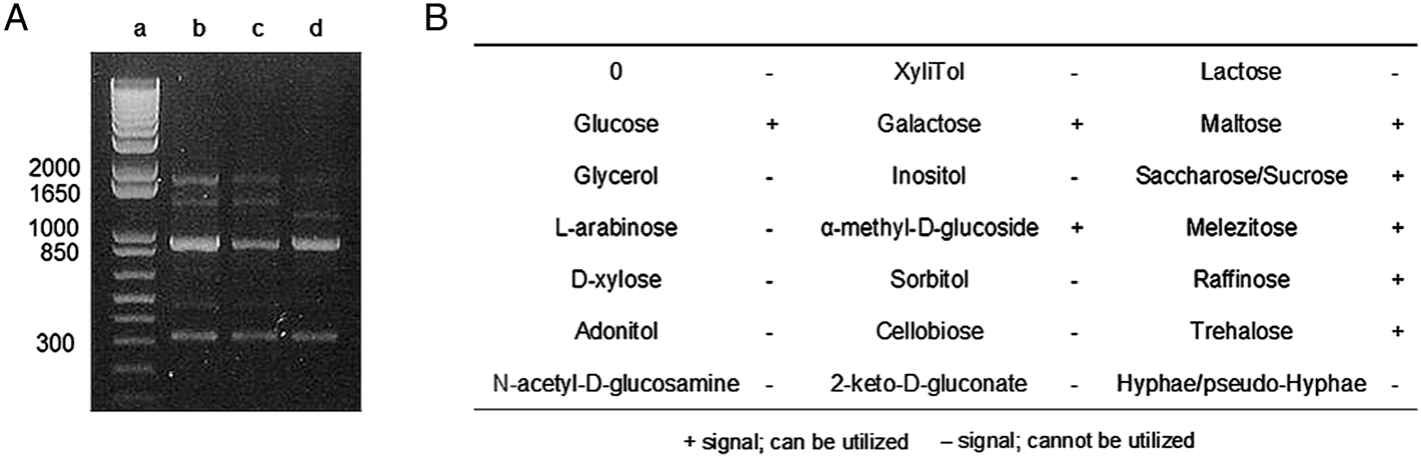
**Identification of** ***Makgeolli*** **yeasts by biomolecular techniques. (A)** RAPD amplification patterns for yeast strains obtained with primer OPA-18. (Lane a) 1 kb marker. (Lane b) Reference strain, *S. cerevisiae* ATCC 9080. (Lane c) Yeast strain in homogenized *Makgeolli* (Lane d) Yeast strain in LHBP-treated *Makgeolli*. **(B)** Carbon source utilization tests for yeast identification in processed *Makgeolli*.

In the present study, the major microorganisms in the *Makgeolli* suspension were rarely inactivated by homogenization treatment with mild pressure (Table 1). In particular, in order to verify a pasteurization effect, target yeasts used as aging indices were monitored based on the thermal death index (here *D*-value) after the LHBP-treatment process. The selected yeast was identified as *S. cerevisiae* with a significance of 99.9% as determined by RAPD monomorphic pattern analysis and a growth promotion test (Figure 5).

**Table 1:** **Thermal resistance index after optimal LHBP process**

**Pretreatment**	**Target microorganisms**				
	**Yeast** ***D*** **values (min)** ^**a**^			**Yeasts (CFU/ml)**	**Bacteria (CFU/ml)**
	***D***_**50.5**_	***D***_**53.5**_	***D***_**57.5**_		
Untreated	-	-	-	~10^7^	~10^6^
Homogenized only (twice at 25.0 MPa)	-	-	-	~10^7^	~10^6^
Pasteurized only	1.06	1.00	0.22	~10^7^ (after 7 days; at *D*_50.5_)	~10^3^ (after 35 days)
				~10^7^ (after 7 days; at *D*_53.5_)	
				~10^7^ (after 14 days; at *D*_57.5_)	
HBP-treated (twice at 25.0 MPa)	0.87	0.63	0.20	~10^7^ (after 7 days; at *D*_50.5_)	10^2^–10^3^ (after 35 days)
				~10^7^ (after 7 days; at *D*_53.5_)	
				< 10^0^ (after 14 day; at *D*_57.5_)	

^a^Thermal death time of *S. cerevisiae*.

**Figure 6:**
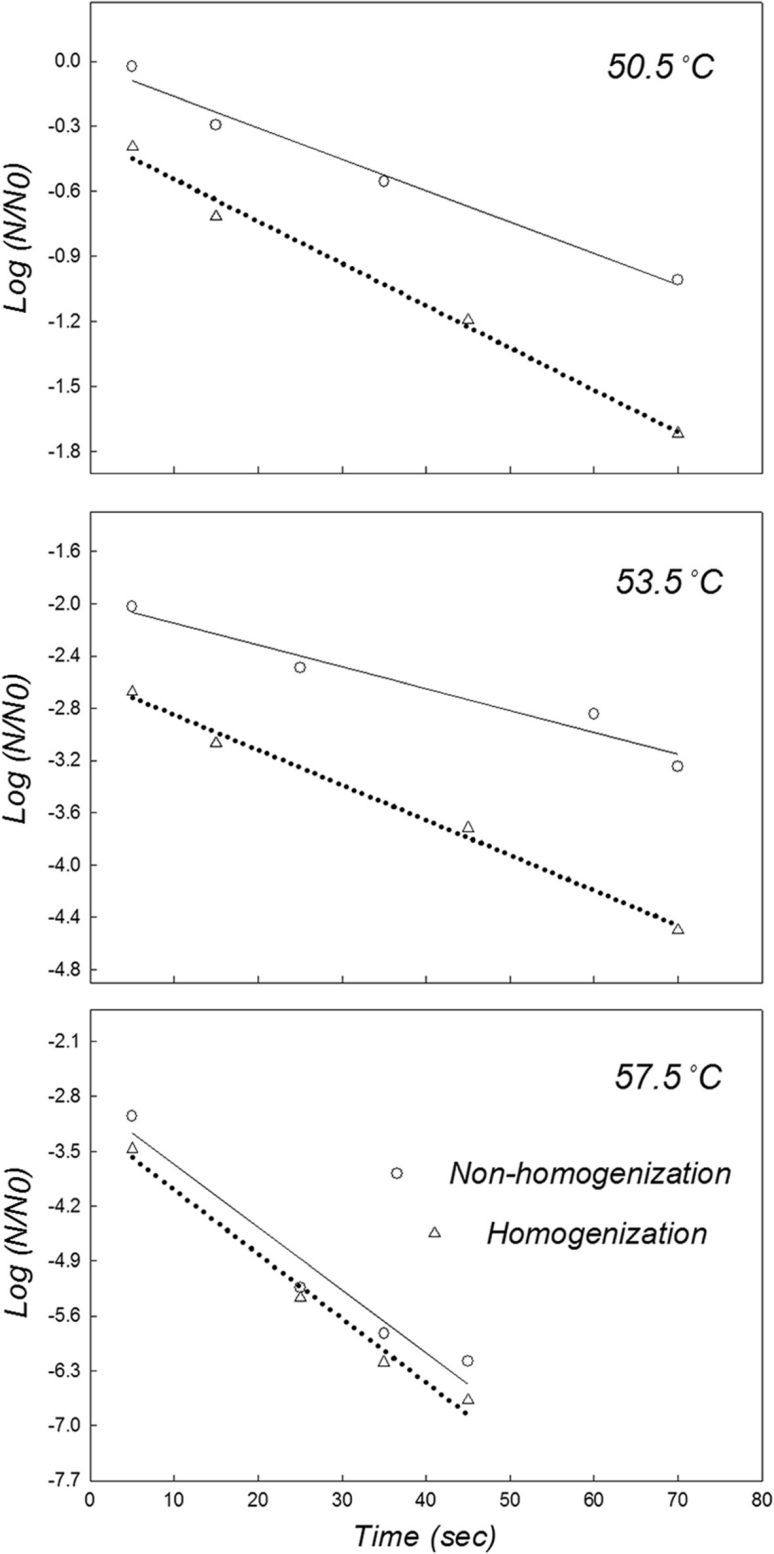
**Effect of LHBP treatment on inactivation of** ***S. cerevisiae*** **strains.** *Makgeolli* samples were applied to the LHBP program based on optimal condition (i.e., homogenized twice at 25.0 MPa). After the LHBP process, the number of target colonies was converted to logarithm (base 10) of colony forming units per gram (log CFU/g). All data shown are the mean values of triplicate observations.

As shown in the colloidal properties of pretreated *Makgeolli* (Figures 1 and 4), the sample that was homogenized twice at 25.0 MPa had the highest dispersion index (i.e., stability) and the lowest flow index (i.e., viscosity) independent of various physicochemical parameters such as temperature, acidity, and time (Table 2). The effect of pasteurization temperature on inactivation of *S. cerevisiae* under the optimal condition of twice 25.0 MPa is shown in Figure 6. The number of surviving *S. cerevisiae* cells was reduced by 3.2 logs following non-LHBP treatment at either 53.5°C (for 70 sec) or at 57.5°C (for 20 sec), while 4.5-log reductions were achieved following LHBP treatment at either 53.5°C (for 70 sec) or 57.5°C (for 16 sec). However, regardless of either LHBP (6.9-log reduction at 57.5°C) or non-LHBP (6.5-log reduction at 57.5°C) treatment, the maximal value of thermal death points did not change significantly with increasing heating times. When compared to the non-LHBP system, especially without homogenization, the synergistic effect of thermal resistance (as *D*-values) were 17.9, 37.7, and 9.1% at 50.5, 53.5, and 57.5°C, respectively (Table 1). Additionally, the level of bacteria maintained the growth limitation for commercial value and continued to maintain a sufficient shelf life without spoilage. These results are in accordance with previous studies that indicate that the inactivation effect of heat treatment on yeasts (whether approximately 0.63–10 min at 55.0–60.0°C or approximately 0.38 min at 80.0°C) and bacteria (approximately 5 min at 55.0–60.0°C) increased concomitantly with pasteurization temperature (Bae et al. 1990; Lee et al. 1991). On the other hand, the advanced LHBP platform has the advantage of saving time and energy (Figure 6) based on the adequate regeneration of insoluble-particles (Figures 2 and 3). This is believed to occur as a result of increased diffusibility of particles and fluidity of intracellular membranes based on adequately controlled pasteurization temperatures (Los and Murata 2004). It is also possible that the close adhesion between a homogeneous solution and the microbial cell membrane might be accelerated by the aggressive deconstruction of aggregated large-molecules.

**Table 2:** **Evaluation of internal quality in** ***Makgeolli*** **before and after optimal LHBP process**

**Pretreatment**	**Key reaction parameters** ^**a**^			
	**Alcohol (%)**	**Acidity**	**Sugar** ^**b**^ **(mg/ml)**	**Amyloglucosidase (U/ml)**
Untreated	~7.0	~3.4	~19.8	~9.0
		~6.3 (after 7 days)	~9.0 (after 7 days)	~8.7 (after 7 days)
Homogenized only (twice at 25.0 MPa)	~7.0	~3.4	~19.9	8.9
		~6.1 (after 7 days)	~8.9 (after 7 days)	~8.8 (after 7 days)
Pasteurized only	~7.0	~3.4	~20.6	~1.4
	~7.0 (after 35 days)	~3.8(after 35 days)	~22.3 (after 35 days)	~2.8 (after 35 days)
HBP-treated (twice at 25.0 MPa)	~7.0	~3.3	~20.8	~1.3
	~7.0 (after 35 days)	~3.5 (after 35 days)	~23.5 (after 35 days)	~3.0 (after 35 days)

^a^Biochemical changes during cold storage at 4°C.

^b^Remained reducing sugars in suspension.

### Commercial evaluation of LHBP-treated *Makgeolli*

The results of internal evaluation of *Makgeolli* before and after the LHBP system are shown in Table 2. A significant decrease in extracellular activity of the target enzyme (here amyloglucosidase for glucose-induced degradation) was observed during the storage period after LHBP treatment. Particularly, under the optimal homogenization condition, the activity of the amyloglucosidasee enzyme in *Makgeolli* showed reversible regulation, above 2.3-fold after 35 days (1.3 U/ml), compared with the initial state. In the case of homogenization treatment with high pressures (Lim et al. 2004), the expression pattern of amyloglucosidase was similarly observed in cereal suspensions, but interestingly its activity was not below that of the LHBP system. This spontaneous phenomenon may affect the rheological properties (especially reducing sugar content) in colloidal suspension, and further enhance the storage stability of microbial suspensions due to the relatively low extracellular activation of starch hydrolysis by amyloglucosidase. However, no significant differences in acidity and alcohol level could be observed in this system during the 35-day storage period.

Regarding the commercial focus of pretreated *Makgeolli*, after the LHBP process, external indices such as apparent colors and flavors were objectively examined based on relative levels of colorimetric intensity and mass chromatograms, respectively (Table 3). In the non-LHBP system, the intensity of several fusel alcohols (e.g., 3-methyl-1-butanols and 2-phenylethanols) significantly increased compared with the optimal LHBP-treated system during storage. These amyl-forms in unpasteurized alcoholic beverages may induce malt-like off-flavors (above 350 μg/ml) (Hazelwood et al. 2008; Selli et al. 2004), however, regardless of either commercial expiration date (approximately 7 days under cold storage) or LHBP treatment, their odor-active activity (below 200 μg/ml) was considered a defect. Furthermore, the level of 2,3-butanediol (i.e., buttery odor substance from citrate fermentation; Bartowsky and Henschke 2004) predominantly increased more in unpasteurized *Makgeolli* than in LHBP-treated *Makgeolli* during the storage period. Additionally, after the LHBP treatment, the increased concentration of ethyl esters (especially ethyl tetradecanoate; above 60 μg/ml) in the aging alcoholic beverage can induce positive effects such as producing a fresh odor during the storage period (Vanderhaegen et al. 2003). Lastly, after heat pretreatment at below 60°C, the slight changes in *Makgeolli* color lightness was caused by external physicochemical factors, specifically, pressure and temperature. Unlike the unstable amino-carbonyl reaction generated by an interactive correlation between pH and temperature (Reynolds 1963), the intramolecular stability, especially protein stability, in LHBP-pretreated *Makgeolli* is thought to be a consequence of the minimal processing approach used, particularly the constant pH [4.2–4.3] and mild-heat temperatures.

**Table 3:** **Objective index evaluation of** ***Makgeolli*** **by optimal LHBP process**

**Condition**	**External quality parameters**							
	**Apparent color** ^**a**^				**Flavor** ^**b**^			
	**L**	**a**	**b**	**ΔE**	**3-methyl-1-butanol**	**2,3-butanediol**	**2-phenylethanol**	**Ethyl tetradecanoate**
					**(ATV** ^**c**^ **: ~300)**	**(ATV** ^**c**^ **: ~150,000)**	**(ATV** ^**c**^ **: ~925)**	**(ATV** ^**c**^ **: ~400)**
Untreated	75.90 ± 0.13^a^	−1.85 ± 0.04^d^	9.27 ± 0.07^a^	-	104.28 ± 16.84	201.05 ± 34.33	133.35 ± 28.16	45.83 ± 15.04
					(after 7 days)	(after 7 days)	(after 7 days)	(after 7 days)
Homogenized only (twice at 25.0 MPa)	69.41 ± 0.04^b^	−1.58 ± 0.01^c^	5.17 ± 0.01^b^	7.68	101.14 ± 11.66	198.55 ± 28.89	122.20 ± 24.56	43.95 ± 13.01
					(after 7 days)	(after 7 days)	(after 7 days)	(after 7 days)
Pasteurized only (at *D*_57.5_)	61.79 ± 0.07^d^	−1.08 ± 0.01^a^	3.18 ± 0.01^d^	15.39	111.25 ± 8.04	138.83 ± 7.44	120.30 ± 10.22	76.62 ± 18.52
					(after 35 days)	(after 35 days)	(after 35 days)	(after 35 days)
HBP-treated (twice at 25.0 MPa and *D*_57.5_)	67.30 ± 0.03^c^	−1.31 ± 0.02^b^	4.32 ± 0.01^c^	9.94	108.02 ± 5.80	119.66 ± 6.24	116.67 ± 8.90	74.10 ± 14.03
					(after 35 days)	(after 35 days)	(after 35 days)	(after 35 days)

^a^Lightness, 0 (black) ~ 100 (white); Redness, −80 (greenness) ~ 100 (redness); Yellowness, −80 (blueness) ~ 70 (yellowness); Color difference, {(L – L_0_)^2^ + (a – a_0_)^2^ + (b – b_0_)^2^}^1/2^. Superscript characters indicate significant difference at *P* < 0.05 by Duncan’s multiple comparison.

^b^Upregulated target odor-active compounds (μg/ml) with significant difference at 0.01 ≤ *P* < 0.05.

^c^Aroma threshold value in water (in parts per billion).

## Conclusions

Based on the minimal processing concept, the LHBP process in turbid *Makgeolli* dispersion provided a benefit to relatively bioactive intramolecular components by limiting the adverse effects (especially overheating by the presence of large insoluble particles) of heat processing, which often negatively affect *Makgeolli* quality. Remarkably, after optimized homogenization pretreatment under the optimum condition of two repetitions at 25.0 MPa, the *S. cerevisiae* population in unpasteurized *Makgeolli* showed a maximal 6.9-log reduction following heat treatment at 53.5°C for 70 sec. Furthermore, based on commercial evaluation tests, we confirmed that the objective sensory quality of LHBP-treated *Makgeollis* is nearly equal to that of unpasteurized (or pasteurized) *Makgeolli*. However, no “conventional programs” (i.e., benchmark platform runs) were examined in the present work.

## Additional file

Additional file 1: **Supplementary Materials and Methods. Figure S1.** Temperature profiles of THE system during continuous pasteurization. All points shown are the mean values of triplicate observations. **Table S1.** Detail profiles of THE system for LHBP process.
