# Supplementary material for: Effective inactivation of Saccharomyces cerevisiae in minimally processed Makgeolli using low-pressure homogenization-based pasteurization
Source: Springerplus. 2015 Apr 2;4:160. doi: 10.1186/s40064-015-0936-4 (PMC4398680; doi:10.1186/s40064-015-0936-4)
Supplement: Supplementary file 1 — Supplementary Materials and Methods. Figure S1. Temperature profiles of THE system during continuous pasteurization. All points shown are the mean values of triplicate observations. Table S1. Detail profiles of THE system for LHBP process. [file 40064_2015_936_MOESM1_ESM.doc]

Additional file 1

Effective inactivation of *Saccharomyces cerevisiae* in minimally processed *Makgeolli* using low-pressure homogenization-based pasteurization

Jin Seop Bak^a,b^

*^a^Department of Chemical and Biomolecular Engineering, KAIST, Daejeon 305-701, Republic of Korea*

*^b^Institute of Advanced Machinery and Design, Department of Mechanical and Aerospace Engineering, Seoul National University, Seoul 151-744, Republic of Korea*

Corresponding Author: Tel.: + 82 10 3159 7789; fax: + 82 42 350 3910

*E-mail address*: jsbwvav7@kaist.ac.kr (J.S. Bak)

Supplementary Materials and Methods

Unless otherwise noted, all starting materials were purchased from commercial suppliers and were used without further purification.

*Substrate-specific regulation for identification*

Substrate utilization test for *Makgeolli* yeasts was checked by the API 20C AUX system (bioMérieux SA) according to the manufacturer's instructions. Confirmation of isolates was analyzed based on APILAB Plus database. For reference, based on a random sampling method, isolated yeast colonies in *Makgeolli* were incubated for 48 h at 25°C on yeast extract-peptone-dextrose broth, and then carried out the test.

*Preparation for extracellular metabolome in colloidal suspension*

In order to analysis exometabolomic odor-active compounds in processed *Makgeolli*, based on previously confirmed protocol (ref. I), after filtration through a 0.2-μm PVDF filter, the supernatant from the *Makgeolli* broths was evaporated to dryness under vacuum with Speed Vac Plus SC110A (Savant Instruments, Holbrook, NY) at 25°C. After drying, all samples were resuspended in 80 μl of methoxyamine hydrochloride mixture (in 2% pyridine) and then incubated for 90 min at 30°C. Next, *N*-Methyl-*N*-(trimethylsilyl)trifluoroacetamide (80 μl/sample mixture) was added to each sample mixture and then maintained for 30 min at 37°C.

GC-MS *operation and ID*

Total ion chromatograms (TICs) using the mass analyzer was performed to analyze the regulation patterns of odor-active compounds on LHBP-treated sample as compared to those of the control. Under the condition of 1 ml He/min, the injection volume was 1 μl, and the split ratio was 1:100. The mass program was operated in SCAN mode (50–550 amu), and further, in order to enhance a quantitative accuracy, all pattern analysis was kept pace with SIM mode. To minimize analytical errors, the area of each peak obtained from the TIC normalized using authentic standards (e.g., 2-ethyl-1-hexanol) were utilized for the quantitative comparison of each compounds (ref. II, ref. III). The m/z values of target compounds (3-methyl-1-butanol, 2,3-butanediol, 2-phenylethanol, and ethyl tetradecanoate) were observed at 70, 57, 91, and 88, respectively.

Supplementary References

1. Bak, J.S., 2015. Lignocellulose depolymerization occurs via an environmentally adapted metabolic cascades in the wood-rotting basidiomycete *Phanerochaete chrysosporium*. Microbiologyopen 4, 151–166.
2. Fiehn, O., Kopka, J., Trethewey, R.N., Willmitzer, L., 2000. Identification of uncommon plant metabolites based on calculation of elemental compositions using gas chromatography and quadrupole mass spectrometry. Anal. Chem. 72, 3573–3580.
3. Bak, J.S., 2015. Extracellular breakdown of lignocellulosic biomass by *Dichomitus squalens*: peroxidation-based platform and homeostatic regulation. Biotechnol. Lett. 37, 349–58.

Supplementary Results

Supplementary Figure Legends

**Figure S1**. Temperature profiles of THE system during continuous pasteurization. All points shown are the mean values of triplicate observations.

Supplementary Table Legends

**Table S1**. Detail profiles of THE system for LHBP process.

**Figure S1**


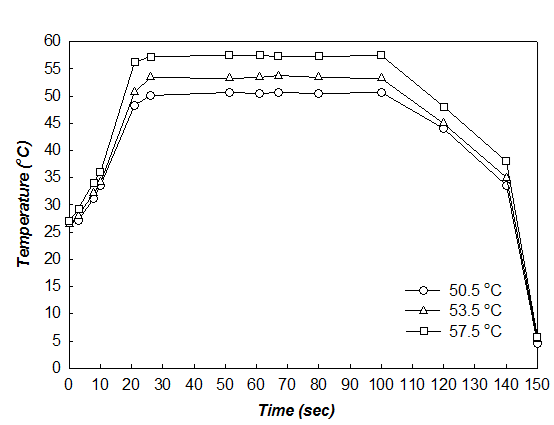


**Table S1. Detail profiles of THE system for LHBP process.**

| Pretreatment condition | Physical parameters of glass tube | | | | Heating time (sec) | Cooling | |
| --- | --- | --- | --- | --- | --- | --- | --- |
|  | *ϕ* (mm) | | Flow rate ^a^  (ml/sec) | Length (cm) |  | Temperature (^o^C) | Time (min) |
|  | Inside diameter | Outside diameter |  |  |  |  |  |
| Untreated | 3.0  3.0  3.0 | 5.0  5.0  5.0 | 1.4 | 1,817 | 5–70 | 4.5 | 1.5 |
| Homogenized once at 15.0, 25.0, and 35.0 MPa |  |  | 1.4 | 1,817 | 5–70 | 4.5 | 1.5 |
| Homogenized twice at 15.0, 25.0, and 35.0 MPa |  |  | 1.4 | 1,817 | 5–70 | 4.5 | 1.5 |

^a^ CX-300 model (Cheonsei Industrial Co., Ltd., Ansan, Korea).
